# Supplementary material for: Comparison of lower limb muscle activation between slow and fast tempos during the stepping test in young men
Source: PLoS One. 2025 Nov 21;20(11):e0337330. doi: 10.1371/journal.pone.0337330 (PMC12637985; doi:10.1371/journal.pone.0337330)
Supplement: Attachment — Submitted filename: Response to Reviewers.doc [file pone.0337330.s002.doc]

Dear reviewers,

We sincerely appreciate the reviewers for their careful and thorough evaluation of our manuscript. Revising it in light of their critiques has been extremely beneficial, and we are confident that their comments have greatly improved both the content and overall quality of our work. Wherever possible, we have incorporated the reviewers’ criticisms, questions, and suggestions to enhance the quality of the manuscript.

Our responses to the comments are presented in the order in which they were raised. Many of our replies required insertions and modifications within the manuscript itself, which are indicated in the revised annotated version with (Page, Line) notation. All changes in the revised manuscript are highlighted, and a list of the revisions is provided together with our point-by-point responses to the reviewers’ comments.

We have also included in this cover letter our confirmations and responses to the Journal Requirements, as well as our replies to both reviewers regarding the revisions. We kindly ask you to review these materials.

We hope that the revised manuscript now meets the requirements for publication in PLOS ONE. We greatly appreciate your consideration.

Sincerely,

------------

Journal Requirements:

When submitting your revision, we need you to address these additional requirements.

1. Please ensure that your manuscript meets PLOS ONE's style requirements, including those for file naming. The PLOS ONE style templates can be found at

https://journals.plos.org/plosone/s/file?id=wjVg/PLOSOne_formatting_sample_main_body.pdf and

https://journals.plos.org/plosone/s/file?id=ba62/PLOSOne_formatting_sample_title_authors_affiliations.pdf

→I have reviewed the instructions and revised the manuscript to comply with the PLOS ONE style requirements.

2. Thank you for stating the following financial disclosure:

K.K. (Keiji Koyama) was supported by a Grant-in-Aid for Scientific Research from the Japan Society for the Promotion of Science (Grant-in-Aid for Young Scientists (19K20021) and Grant-in-Aid for Scientific Research (23K10674)). Please state what role the funders took in the study. If the funders had no role, please state: "The funders had no role in study design, data collection and analysis, decision to publish, or preparation of the manuscript."

If this statement is not correct you must amend it as needed. Please include this amended Role of Funder statement in your cover letter; we will change the online submission form on your behalf. 「The funders had no role in study design, data collection and analysis, decision to publish, or preparation of the manuscript.」

→The funders were not involved in the study design, data collection and analysis, decision to publish, or preparation of the manuscript. Therefore, the statement “The funders had no role in study design, data collection and analysis, decision to publish, or preparation of the manuscript. ” is correct. I would appreciate it if you could make this change. I would be grateful if you could update the online submission form accordingly.

3. Thank you for stating the following in the Competing Interests section:

The authors declare that they have no known competing financial or personal interests that could have appeared to influence the work reported in this paper. Please confirm that this does not alter your adherence to all PLOS ONE policies on sharing data and materials, by including the following statement: "This does not alter our adherence to PLOS ONE policies on sharing data and materials.” (as detailed online in our guide for authors http://journals.plos.org/plosone/s/competing-interests). If there are restrictions on sharing of data and/or materials, please state these. Please note that we cannot proceed with consideration of your article until this information has been declared. Please include your updated Competing Interests statement in your cover letter; we will change the online submission form on your behalf.

→I have confirmed this. Please add the following statement to the Competing Interests section: "This does not alter our adherence to PLOS ONE policies on sharing data and materials.” I would be grateful if you could update the online submission form accordingly.

4. In the online submission form, you indicated that the data that support the findings of this study are not publicly available due to ethical restrictions, but are available from the corresponding author upon reasonable request. All PLOS journals now require all data underlying the findings described in their manuscript to be freely available to other researchers, either a. In a public repository, b. Within the manuscript itself, or c. Uploaded as supplementary information. This policy applies to all data except where public deposition would breach compliance with the protocol approved by your research ethics board. If your data cannot be made publicly available for ethical or legal reasons (e.g., public availability would compromise patient privacy), please explain your reasons on resubmission and your exemption request will be escalated for approval.

→I have reviewed the requirement. All data underlying the findings of this study are already provided within the manuscript, and thus comply with the PLOS ONE data availability policy. I have also updated the information in the online submission form when submitting the revised manuscript, and I would appreciate it if the editorial office could kindly confirm this on your end as well.

5. If the reviewer comments include a recommendation to cite specific previously published works, please review and evaluate these publications to determine whether they are relevant and should be cited. There is no requirement to cite these works unless the editor has indicated otherwise.

→Thank you for your suggestion. I have carefully evaluated the recommended references and revised the manuscript as appropriate

---------------

Review Comments to the Author

Reviewer #1: General Comments:

Overall, this is a good manuscript that presents how stepping tempo affects lower limb muscle activation and dynamic balance in young men, adding useful insight for rehabilitation and training contexts. The study design is methodologically solid, with a clear rationale and appropriate EMG and force plate measures. The introduction reviews the relevant biomechanical literature well but could frame a sharper hypothesis. The methods are described in sufficient detail for replication, and the results are clearly presented with appropriate stats and effect sizes. The discussion interprets the biomechanical implications accurately, comparing them to relevant previous studies (e.g., Shin & Demura, Lux et al.), but would benefit from deeper linkage to comparable EMG literature and clearer acknowledgment of limitations. Overall, the paper is well-structured, but there is room for improvement, specifically with the discussion.

→Thank you for your valuable comments. Based on your suggestions, I have revised the manuscript, particularly the Discussion section. I would appreciate it if you could review the revised version.

Specific Comments:

- Abstract: Consider including specific results or quantitative outcomes in the abstract. Currently it states the general findings, but adding some numbers (e.g., percentage reductions in EMG, etc) make it more clear.

→Retouched >>>(Page 2, Line 23-26)

- L45–109 (Introduction): The introduction is well-written and references relevant stepping, balance, and EMG studies (e.g., Shin & Demura). However, it does not explicitly state a hypothesis. Recommend adding a sentence to clarify the expectation: e.g., "It was hypothesized that stepping at slower tempos would increase integrated muscle activation due to prolonged single-leg stance."

→Retouched >>>(Page 4, Line 96 to Page 5, Line 108)

- L163–180 (Experimental Procedure): The force plate setup is described clearly, but specify if two force plates were used simultaneously for bilateral data or if only the right leg was analyzed. Similar studies (Lux et al., 1992) analyzed bilateral data.

→Retouched >>>(Page 7, Line 168 and Line 175-179)

- L182–194 (Time Parameters): Only the right leg’s single-leg support is analyzed — explain why you chose the right leg only. If you assume limb symmetry, justify it.

→Retouched >>>(Page 7, Line 185-189)

- L204–222 (Vertical GRF): the filter description is awkwardly worded. Consider rewriting for clarity: “Signals were low-pass filtered at 20 Hz using a zero-phase-lag FIR filter with a Kaiser window…”

→Retouched >>>(Page 9, Line 229)

- Also, clarify if vertical only or other force components were measured

→Retouched >>>(Page 8, Line 220)

- — as mediolateral forces could affect balance.

→(Page 19, Line 550-553)

- L232–279 (EMG Analysis): clarify whether the electrode placement followed SENIAM guidelines.

→Retouched >>>(Page 10, Line 265)

- Also, indicate whether crosstalk between adjacent muscles was considered.

→Retouched >>>(Page 10, Line 272)

- L281–296 (Data Analysis): It would strengthen the text to mention whether you controlled for multiple comparisons formally.

→Retouched >>>(Page 11, Line 310-316)

- Also, you might clarify whether assumptions for parametric tests were checked.

→Retouched >>>(Page 12, Line 318-323)

- L298–337 (Results): The results are clear and effect sizes provided. The large standard deviations for EMG suggest high inter-individual variability — discuss this more in the Discussion (which you partly do).

→Added and retouched>>>(Page 18, Line 505-523)

- L343–354 (Discussion): The main finding is correctly interpreted: slow tempo increases balance demand and integrated EMG. Connect this more directly to studies like Shin & Demura (2007, 2009) and Lux et al. (1992) who found greater quadriceps activation in step-up exercises. This strengthens your comparison to prior EMG literature.

→Added and retouched>>>(Page 14, Line 412-425)

- L355–394: you mention that muscles like the tibialis anterior and peroneals may play a role — you should note that these muscles were not recorded. Recommend clarifying this as a limitation.

→Added>>>(Page 17, Line 461-468)

→Added>>>(Page 20, Line 553-557)

- L411–434: The explanation of impact force differences is logical, but it would help to connect this to landing mechanics literature (e.g., foot strike patterns in running). You do this later — consider merging or cross-referencing these points.

→Retouched>>>(Page 18, Line 505-Page 19, Line 523)

- L437–451: You acknowledge large SDs and inter-individual variability well. Consider suggesting that a motion capture analysis could confirm joint angle influences.

→Added>>>(Page 19, Line 533-535)

- L455–468 (Limitations): add that you did not record trunk EMG, which could contribute to mediolateral stability, as prior studies suggest (e.g., Kaur et al., 2022).

→Added>>>(Page 16, Line 455-461)

→Added>>>(Page 20, Line 553-557)

- Also, your subjects are young adults, but the study's findings are more applicable to the older population. You should clarify and explain this extensively in the limitations.

→Added>>>(Page 20, Line 557-563)

Reviewer #2: General comment

This study addresses an important question regarding which lower limb muscles contribute to dynamic balance during a stepping test. However, the main conclusion – that “maintaining dynamic balance requires force generation from all lower limb muscles rather than a specific one” – requires more cautious interpretation based on the data presented. I recommend considering the following points for revision.

→Thank you very much for your valuable comment. As you pointed out, it is difficult to determine from our data whether the maintenance of dynamic balance during stepping in young adults is attributable to force generation from all lower limb muscles or to the prolonged single-leg support time. Therefore, we have revised the discussion and conclusion to provide a more cautious interpretation based on the results. We kindly ask you to review the revised version.

#Major comments

1. Interpretation of iEMG values

iEMG is strongly influenced by movement duration, which may partly explain the larger values observed at the slow tempo. It seems self-evident that iEMG would be greater in the slow condition due to the longer step duration. Therefore, please clarify the rationale for using iEMG

→Added>>>(Page 4, Line 96-Page 5, Line 108)

and explain what new insights were gained from comparing it with mEMG.

→Added>>>(Page 17, Line 470-Page 18, Line 501)

2. Validity of using metronome synchronization error as a balance index

If the metronome tempo is constant, participants may be able to maintain a steady stepping rhythm even without auditory cues. Thus, the time difference between foot contact and the metronome sound may not fully reflect dynamic balance ability. Although you referenced previous studies for using this metric, please provide additional justification for its validity in this context. For instance, would introducing random tempo variations improve the reliability of this measure as a balance index?

→Retouched>>>(Page 14, Line 392-Page 15, Line 410)

→Added>>>(Page 15, Line 412-Page 16, Line 438)

3. mEMG findings contrary to the hypothesis

The mEMG values were higher in the fast tempo condition, which contradicts the hypothesis that muscle activity would increase under the more unstable slow tempo condition. Please provide additional discussion on why this might have occurred.

→Added>>>(Page 17, Line 470-Page 18, Line 501)

4. Interpretation of single-leg support time

The longer single-leg support time at the slow tempo seems like an expected outcome due to slower stepping. Please clarify the significance of presenting and statistically testing this result. How does this difference meaningfully contribute to evaluating dynamic balance ability?

→Added>>>(Page 15, Line 401-410)

→Added>>>(Page 15, Line 412-417)

5. Practical significance of time difference

Although the time difference between foot contact and metronome sound was statistically different between tempos, its functional or clinical relevance is unclear. Please discuss the practical implications of this difference.

→Added>>>(Page 14, Line 391-Page 15, Line 410)

→Added>>>(Page 15, Line 427-Page 16, Line 438)

→Added>>>(Page 19, Line 544-549)

6. Statistical analysis description

The manuscript states that “measurement variables (single-leg support time, time difference, ground reaction force variables, %mEMG, and %iEMG) were used as dependent variables, and tempo (slow/fast) as an independent variable for paired t-tests.” This description is inappropriate because paired t-tests do not use the framework of dependent and independent variables. Please revise this statement for accuracy.

→Retouched>>>(Page 12, Line 318-323)

#Minor comments

7. Figure 1 resolution and labeling

The resolution of Figure 1 is low, making it difficult to read. Additionally, the figure lacks legends for the rectangular and continuous signals. I assume the rectangular signal indicates metronome timing, but is its width precisely aligned with the metronome interval? If the continuous signal represents ground reaction force (with a 10 N threshold), please indicate the exact points of foot contact and toe-off in the figure.

→Changed Figure 1

→Retouched>>>(Page 8, Line 212-217)

8. Raw vs. processed data in Figure 2

Figure 2 seems to illustrate data processing for ground reaction forces. It would improve transparency if you also provide raw and processed signals for both ground reaction forces and EMG to allow readers to evaluate the effect of filtering.

→Changed Figure 2

→Retouched>>>(Page 9, Line 247-250)

---------------
