# Supplementary material for: Comparison of lower limb muscle activation between slow and fast tempos during the stepping test in young men
Source: PLoS One. 2025 Nov 21;20(11):e0337330. doi: 10.1371/journal.pone.0337330 (PMC12637985; doi:10.1371/journal.pone.0337330)
Supplement: Attachment — Submitted filename: R2_Response to Reviewers.doc [file pone.0337330.s003.doc]

Dear reviewers,

Following the first revision, we would like to express our sincere gratitude once again for your careful and thorough evaluation of our manuscript. Revising the paper in light of your comments has again been extremely valuable, and we are confident that your feedback has further improved both the content and the overall quality of the manuscript. We have carefully reviewed all of your critiques, questions, and suggestions, and have incorporated them wherever possible to enhance the completeness and clarity of the paper.

Our responses are presented in the order in which the comments were provided. Many of our replies correspond to insertions or revisions made directly in the manuscript, with the relevant locations indicated by page and line numbers in the newly annotated version. All modifications are highlighted in the revised manuscript, and a detailed list of the changes, together with our point-by-point responses to each reviewer’s comments, is included. In addition, this cover letter summarizes the main revisions made in response to the reviewers’ feedback. We kindly ask you to review these materials.

We hope that the revised version of the manuscript now meets the requirements for publication in PLOS ONE.

Thank you very much for your continued time and consideration.

Sincerely,

------------

Reviewer #2:

We sincerely appreciate your continued careful review of our manuscript and your insightful comments. In particular, your remarks on “#Validity of the metronome synchronization error & clinical significance of the time difference” were extremely valuable and prompted us to reconsider part of our interpretation regarding the time difference results. We are truly grateful for your constructive feedback. The specific revisions and their corresponding locations in the revised manuscript are provided below for your reference.

#Validity of the metronome synchronization error & clinical significance of the time difference

In this study, the time difference between metronome sound and foot contact is used as an indicator of “dynamic balance.” However, I have concerns about presenting this measure as one of the main results. The reason is that the chosen tempos (44 bpm and 132 bpm) are relatively fast, and especially at 132 bpm, movement speed and reaction time constraints are likely to strongly influence timing control. Thus, caution is warranted before concluding that this synchronization error directly reflects dynamic balance. Rather, it may be more appropriate to interpret it as an index of rhythm synchronization ability or motor timing accuracy.

→Added >>>(Page 15, Lines 425–442)

Furthermore, although the difference was statistically significant between tempos, the functional and clinical implications of the magnitude of this difference remain unclear. At present, I believe it is more appropriate to regard it as an auxiliary timing index that may indirectly relate to balance, rather than as a primary balance indicator. Since the main objective of this study is to examine changes in lower limb muscle activity under different tempo conditions, the synchronization error analysis should be positioned as a supplementary result, with its limitations clearly stated. I leave it to the editor to decide whether and how the authors should further address this issue or whether the manuscript is acceptable in its current form.

→Added >>>(Page 20, Lines 566–568)

→Changed and modified >>>(Page 21, Lines 586–600)

#Figure 1 (resolution and labeling)

For Figure 1, the labeling has improved; however, the resolution itself does not appear to have been sufficiently enhanced. I would leave it to the editorial office to judge whether the resolution is adequate for publication.

→Improved the resolution of Figure1 and Figure 2.

------------
