# Supplementary material for: Comparison of lower limb muscle activation between slow and fast tempos during the stepping test in young men
Source: PLoS One. 2025 Nov 21;20(11):e0337330. doi: 10.1371/journal.pone.0337330 (PMC12637985; doi:10.1371/journal.pone.0337330)
Supplement: Attachment — Submitted filename: R3_Response to Reviewers.doc [file pone.0337330.s004.doc]

Dear Dr. Emiliano Cè,

Following the second revision, we would like to sincerely thank you once again for your valuable comments. We have made the necessary revisions to our manuscript (PONE-D-25-30664R2), entitled “Comparison of lower limb muscle activation between slow and fast tempos during the stepping test in young men.”

Regarding the comment from Reviewer #2, the only remaining issue concerned the image resolution. To address this, we used PLOS’s free online tool NAAS to verify and adjust the figures to meet the publication-quality standards. Both Figure 1 and Figure 2 were confirmed by NAAS to fully comply with PLOS ONE’s figure specifications. In addition, we confirmed that when viewing the PDF version of the manuscript, clicking the “Click here to access/download” link in the upper right corner of each figure opens the original image, which appears clear and high in resolution.

As Reviewer #2 kindly mentioned, the evaluation of image resolution should be handled by the editor and the editorial office. Therefore, we would greatly appreciate it if you could review the figures and confirm that they meet the journal’s requirements. If there are still any issues with the resolution of Figures 1 or 2, please kindly let us know.

As mentioned above, since the revision concerned only the figure resolution and no changes were made to the manuscript text itself, the “Manuscript” and “Revised Manuscript with Track Changes” files are identical to those submitted in the second revision. Therefore, there are no highlighted modifications in the tracked-changes version.

Thank you very much for your continued consideration.

Warm regards,

Keiji Koyama

Keiji Koyama, Ph.D.

Department of Sport Science

Toin University of Yokohama

1614 Kurogane, Yokohama-shi, Kanagawa 225-8503, JAPAN

E-mail: [koyakei@toin.ac.jp](mailto:koyakei@toin.ac.jp)
